# Comparative study between fractional CO2 laser alone versus fractional CO2 laser combined with topical dutasteride in treatment of male androgenic alopecia

**DOI:** 10.1007/s10103-024-04269-8

**Published:** 2025-01-11

**Authors:** Sara Ahmed Galal, Mona Sobh Ali, Hala Shawky A. HafizHala

**Affiliations:** Present Address: Dermatology and Venereology Department, Faculty of Medicine (Girls), Al-Azhar University, 53, New Cairo, 3rd Zone Fifth, Settlement, Cairo, Egypt

**Keywords:** Androgenic alopecia, Fractional CO 2 laser, Dutasteride

## Abstract

Androgenic alopecia (AGA) is the most common form of non-scarring hair loss, characterized by marked hair follicle miniaturization. AGA is a challenging skin condition with limited treatment results. Laser light can promote hair growth at specific wavelengths. The efficacy of fractional CO2 laser in scalp AGA treatment was reported in a few studies. We aimed to compare the efficacy of fractional CO2 laser alone versus the combination of fractional CO2 laser with topical dutasteride in the treatment of male AGA. 30 male patients with AGA were enrolled in the study; they were divided into two groups. All patients received three sessions of ablative fractional CO2 laser one month apart on the scalp, where group (I) patients were subjected to laser sessions only, and group (II) patients were subjected to topical dutasteride, first immediately after each session and secondly fifteen days after each session. The evaluation was done according to dermoscopy (DermLite^®^ DL4) and photographic assessment. Patient satisfaction and side effects were reported. According to the global photo assessment, the combination of fractional CO2 laser with topical dutasteride showed a statistically significant improvement compared to the fractional CO2 laser alone group. The combination of fractional CO2 laser with topical dutasteride is more efficient in improving male androgenic alopecia than fractional CO2 laser alone according to the investigator’s global assessment. There was a significant improvement in all dermoscopic parameters in both groups.

## Introduction

Male androgenetic alopecia (AGA) is a condition characterized by scalp hair thinning [1]. It has a psychological effect on the quality of life and affects about 70% of men [2].

AGA is a multifactorial skin disease with a complex genetic background [1] in which there is a disturbance of androgen signaling resulting in a gradual decrease in the anagen phase and a delay in the telogen-to-anagen transition and follicular miniaturization [3].

The pathogenesis of male pattern baldness also requires the existence of the testosterone hormone. Testosterone is converted to dihydrotestosterone, which is a more potent androgen, by the 5-α-reductase enzyme [4].

There are many current treatment modalities to enhance hair growth, including minoxidil or finasteride; however, there is no effective treatment yet for this challenging condition [5].

Finasteride, an approved drug for AGA treatment, is a type 2 5-alpha reductase (5AR) inhibitor. Dutasteride is a synthetic 4-azasteroid. It is a selective competitive inhibitor of type-1 and type-2 5AR isoenzymes [1]. Dutasteride is three times and 100 times more potent than finasteride in inhibiting type 2 5AR and type 1 5AR, respectively [6].

Fractional CO2 and erbium glass have been studied as treatment choices for AGA. The CO2 lasers emit light at a wavelength of 10,600 nm. Both CO2 and Erbium glass lasers chromophore are water, where they both effectively coagulate or vaporize tissue. Due to evidence that certain wavelengths of laser light encourage hair growth, using lasers for hair loss treatment has become promising [7].

The fractional CO2 laser can be also used to assist drug delivery and enhance penetration and the homogenous distribution of topically applied drugs, which improves drug delivery and bioavailability [8].

Initial evidence suggests that fractional CO2 laser therapies have a promising effect on hair regrowth [5]. A few studies suggest the role of fractional lasers in hair regrowth. To our knowledge, this is the first study to evaluate the effect of fractional CO2 laser with topical dutasteride in the treatment of male AGA.

Accordingly, we aimed to evaluate the effect of fractional CO2 laser alone versus fractional CO2 laser with topical dutasteride in the treatment of AGA.

## Patients & methods

This prospective comparative study was conducted on 30 male patients complaining of AGA. Cases were recruited from the outpatient clinic from October 2021 until December 2022. Informed written consent was obtained from all participants. Approval from the Research Ethics Committee of the Faculty of Medicine of the university was also obtained.

### Patients

Male patients complaining of AGA were included in this study. The participants were informed about the aim and methods of the study.

Females were excluded from the study, as fractional CO2 lasers can lead to hair cutting during the session. We excluded patients under the age of 18 and patients with other dermatological conditions, chronic systemic diseases, or those taking medications that can affect hair. Patients with active infections, a history of keloid, and patients receiving any line of treatment to promote hair growth during the past 3 months were also excluded.

### Treatment protocol

All patients were subjected to a full history, general examination, and dermatological examination. A history of previous treatment for AGA, if present, will be obtained. The patients were carefully evaluated and graded according to the Norwood-Hamilton classification into seven grades [9].

Photographs were taken by the Infinix X601-LTE phone`s camera (13 megapixels) before each session and 3 months after the last session.

Dermoscopic evaluation using a polarized light dermoscopy (Derm Lite, 3 Gen, USA) with a magnification of 10-fold connected to an Infinix X601-LTE phone`s camera was done. The dermoscopic signs of AGA include the percentage of vellus hair, terminal hair, hair diameter diversity, peripilar sign, and yellow dots [10].

### Technique

The patients were instructed to wash their heads and not apply any topical agents to the scalp. All patients received three sessions of ablative fractional CO2 laser (SmartXide DOT DEKA, Florence, Italy) one month apart on their scalp using the following parameters: in DOT fractional scanning mode, the output power was flexible and chosen from 10 to 20 W based on the density of the scalp hair. A scanning dual time of 400 µs, dot spacing of 400 μm, one stack, square shape, ratio 10/10, and size 100% with one pass done to the treated area. These parameters were equivalent to fluence ranging from 0.71 J/cm² to 1.42 J/cm², pulse energy X DOT ranging from 4.0 mJ to 8.0 mJ, and coverage density of 17.1%.

The patients were advised to avoid sun exposure and not apply any other topical medication after the session.

The patients were divided into two groups. The first group (15 patients) was treated with laser sessions only. The second group (15 patients) was treated with 1 ml of topical dutasteride (0.002%) applied immediately after each laser session.

### Assessments

The patients were evaluated at baseline, before each session, and 3 months after the last session. The responses were assessed according to:

The investigator’s global assessment [IGA]: It was done by the researchers and two blinded dermatologists as follows: excellent improvement; +3 points, good improvement; +2 points, poor improvement; +1 point, no change; 0 points, slight deterioration; -1-point, moderate deterioration; -2 points, significant deterioration; -3 points [7].

Dermoscopic evaluation: to detect the signs of AGA (the percentage of vellus hair, terminal hair, hair diameter diversity, peripilar sign, and yellow dots) and signs of improvement.

Patients’ assessment and satisfaction: After the follow-up period, patients were asked about their degree of satisfaction according to the following: 0 = not satisfied, 1 = poor satisfaction, 2 = moderately satisfied, and 3 = very satisfied. Patients were assessed clinically and asked to report any adverse effects related to the procedure, such as erythema, pain, headache, dandruff, dryness, itching, and broken hair shafts, if present.

### Statistical methods

Data were coded and entered using the statistical package for the Social Sciences (SPSS) version 28 (IBM Corp., Armonk, NY, USA). Data were summarized using mean, standard deviation, median, minimum, and maximum in quantitative data and frequency (count) and relative frequency (percentage) for categorical data.

Comparisons between quantitative variables were done using the non-parametric Kruskal-Wallis and Mann-Whitney tests. For the comparison of paired measurements within each patient, the non-parametric Wilcoxon signed-rank test was used. For comparing categorical data, the chi-square test was performed. An exact test was used instead when the expected frequency was less than 5. For the comparison of serial measurements within each patient, the non-parametric McNemar test was used. Correlations between quantitative variables were done using the Spearman correlation coefficient. P-values less than 0.05 were considered statistically significant.

## Results

The present study included 30 males with AGA with ages ranging from 22 to 45 years.

The grading of AGA using the Norwood-Hamilton classification for patients in each treatment group was demonstrated. In the laser-only group, grade I was found in 2 patients (13.3%), grade II in 2 patients (13.3%), grade III in 4 patients (26.7%), grade IV in 3 patients (20.0%), grade V in 2 patients (13.3%), and grade VI in 2 patients (13.3%). While in the laser plus topical dutasteride group, grade I was found in one patient (6.7%), grade II (6.7%), grade III in 7 patients (46.7%), grade IV in 2 patients (13.3%), grade V in 3 patients (20.0%), and grade VI in one patient (6.7%). No statistically significant difference was found between both groups regarding the grading of AGA (*p* = 0.839) (Table 1).

**Table 1:** Demographic and descriptive data of the participants

	Laser only group						Laser Plus Topical Dutasteride group					*P* value
	Mean		SD	Median	Minimum	Maximum	Mean	SD	Median	Minimum	Maximum	
**Age**	31.13		7.76	31.00	22.00	42.00	32.00	7.22	31.00	22.00	45.00	0.683≠
**Duration**	6.93		4.40	7.00	2.00	14.00	7.13	4.16	6.00	3.00	15.00	0.744≠
**Family history**					**Count**		**%**		**Count**	**%**	**P value**	
** +ve**					**12**		80.0%		15	100.0%	0.224*	
** -ve**					3		20.0%		0	0.0%		
					**Laser only group**		**Laser Plus Topical dutasteride group**				**P value**	
					**Count**	**%**	**Count**		**%**			
**Grade**		**I**			2	13.3%	1		6.7%		0.839*	
		**II**			2	13.3%	1		6.7%			
		**III**			4	26.7%	7		46.7%			
		**IV**			3	20.0%	2		13.3%			
		**V**			2	13.3%	3		20.0%			
		**VI**			2	13.3%	1		6.7%			

P-value > 0.05: Non significant P-value < 0.05: Significant P-value < 0.01: Highly significant

≠: Kruskal-Wallis test; *: Chi-square test

No statistically significant difference was found between both treated groups regarding age, duration of AGA, and family history of AGA as shown in Table 1.

In the laser-only group, there was a significant improvement in all dermoscopic parameters compared to the baseline, the vellus hair decreased from 28.74 ± 22.91 to 24.97 ± 18.55, the hair diameter diversity decreased from 45.45 ± 13.93 to 38.81 ± 13.5, and the single pilosebaceous unit decreased from 99.13 ± 1.16 to 97.85 ± 2.40 with a statistically significant difference (p-value < 0.05). The terminal hair increased significantly from 67.40 ± 22.58 to 72.15 ± 22.86 after treatment (p-value = 0.02). Peripilar signs showed a statistically significant reduction after treatment (found in 6.7% of patients after treatment compared to 13.3% of patients before treatment) (Table 2).

**Table 2:** Comparison of dermoscopic features before and after treatment with Laser Only

Fractional CO2 laser group															
Laser only group	Before							After							
	Mean	SD		Median	Minimum		Maximum	Mean	SD	Median		Minimum	Maximum		*P* value‡
**V**	28.74	22.91		18.50	0.00		67.10	24.97	18.55	17.40		6.30	60.00		0.023
**T**	67.40	22.58		81.50	32.90		88.50	72.15	22.86	82.50		23.10	93.70		0.020
**HDD**	45.45	13.93		48.50	23.80		80.00	38.81	13.50	35.80		21.40	76.90		0.009
**SPSU**	99.13	1.16		100.00	97.10		100.00	97.85	2.40	98.40		90.70	100.00		0.002
**Laser only**					**Before**			**After**						P value*	
					**Count**	**%**		**Count**			**%**				
**PPS**			**+ve**		2	13.3%		1			6.7%			0.001	
			**-ve**		13	86.7%		14			93.3%				
**YD**			**+ve**		10	66.7%		10			66.7%			1	
			**-ve**		5	33.3%		5			33.3%				
**BD**			**+ve**		0	0.0%		0			0.0%			----	
			**-ve**		15	100.0%		15			100.0%				

Vellus hair (V), terminal hair (T), hair diameter diversity (HDD2), single pilosebaceous unit (SPSU), peripilar sign (PPS), yellow dot (YD), and black dot (BD)

P-value > 0.05: Non-significant; P-value < 0.05: Significant; P-value < 0.01: Highly significant

‡: Mann-Whitney test *: Chi-square test

In the laser plus topical dutasteride group, there was a significant improvement in all dermoscopic parameters compared to the baseline, the vellus hair decreased from 22.37 ± 8.53 to 14.50 ± 6.95, hair diameter diversity decreased from 40.95 ± 7.47 to 31.15 ± 6.71, and the single pilosebaceous unit decreased from 97.33 ± 1.72 to 94.33 ± 3.32 with a statistically significant difference (p-value < 0,05). The mean terminal hair showed a statistically significant increase from 78.40 ± 9.08 to 85.23 ± 7.13 after treatment (p-value = 0.005). Peripilar signs showed a statistically significant reduction after treatment (found in 13.3% of patients after treatment compared to 26.7% of patients before treatment) (Table 3).

**Table 3:** Comparison of dermoscopic features before and after treatment with Fractional CO2 laser Plus Topical Dutasteride

Fractional CO2 laser Plus Topical dutasteride group																
	Before							After								
	Mean		SD	Median	Minimum	Maximum		Mean		SD	Median		Minimum	Maximum		*P* value‡
**V**	22.37		8.53	18.90	11.80	42.20		14.50		6.95	13.80		5.00	29.80		0.003
**T**	78.40		9.08	81.80	57.80	89.80		85.23		7.13	86.20		70.20	95.00		0.005
**HDD**	40.95		7.47	37.00	33.70	56.80		31.15		6.71	27.70		22.30	45.30		0.001
**SPSU**	97.33		1.72	97.70	94.80	100.00		94.33		3.32	96.20		89.50	98.60		0.001
**Laser Plus Topical Dutasteride**					**Before**				**After**						P value*	
					**Count**		**%**		**Count**			**%**				
**PPS**		**+ve**			4		26.7%		2			13.3%			0.013	
		**-ve**			11		73.3%		13			86.7%				
**YD**		**+ve**			2		13.3%		3			20.0%			0.317	
		**-ve**			13		86.7%		12			80.0%				
**BD**		**+ve**			0		0.0%		0			0.0%			----	
		**-ve**			15		100.0%		15			100.0%				

Vellus hair (V), terminal hair (T), hair diameter diversity (HDD), single pilosebaceous unit (SPSU), peripilar sign (PPS), yellow dot (YD), and black dot (BD)

P-value > 0.05: Non-significant; P-value < 0.05: Significant; P-value < 0.01: Highly significant

‡: Mann-Whitney test *: Chi-square test

On comparing the improvement of dermoscopic signs between both groups after treatment, no statistically significant difference was found except for a single pilosebaceous unit that showed a statistically significant reduction in the laser plus topical dutasteride group compared to the laser-only group (p-value < 0.001) (Table 4).

**Fig. 1:**
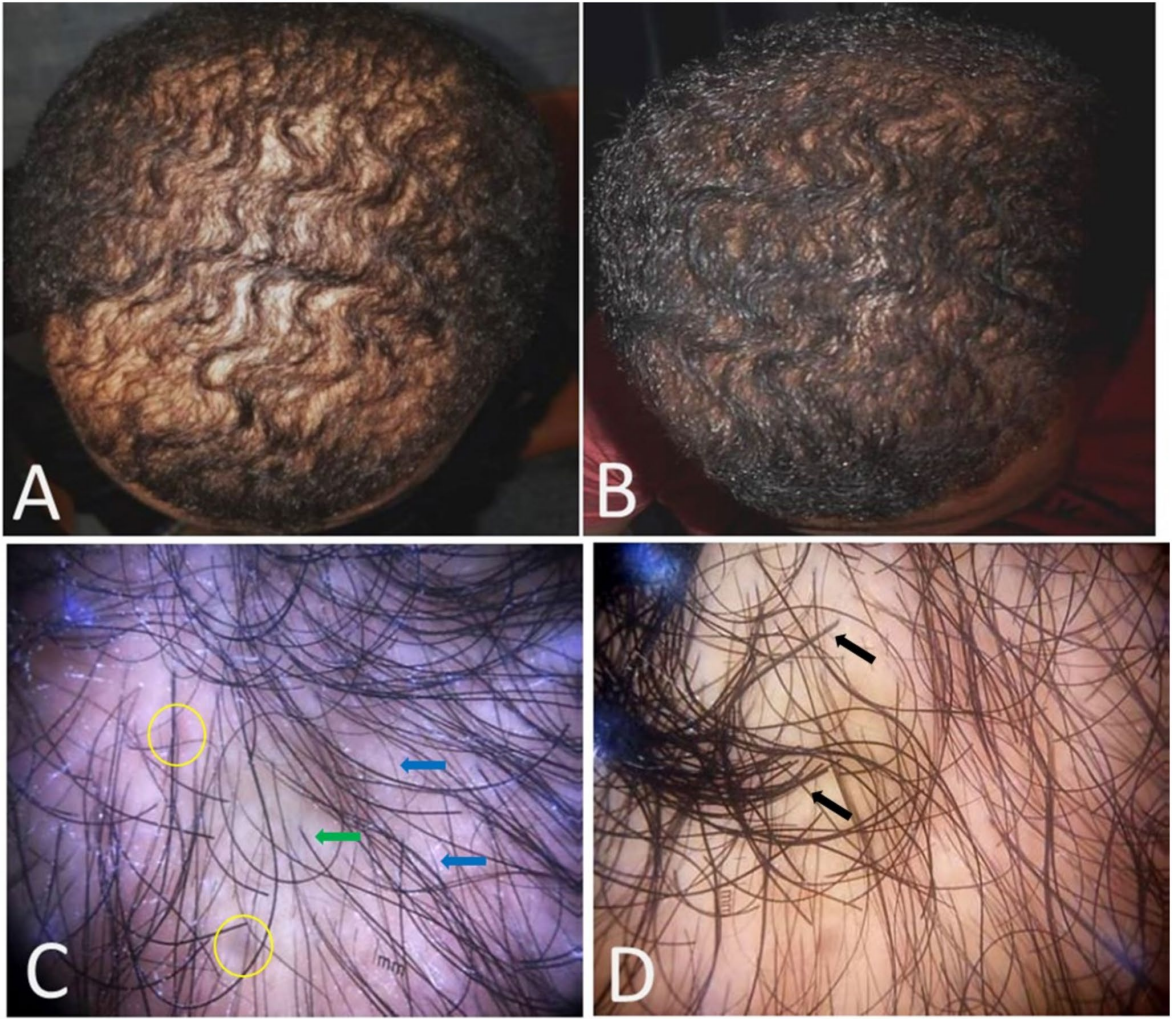
26-year-old male patient, grade II AGA, with 3-year duration (**A**) Clinical photos before treatment (**B**) showing moderate improvement after treatment. (**C**) dermoscopic photos before treatment, counting vellus hair (11.8%), terminal hair (88.2%), and calculating hair diameter diversity (41.7%) per field. (**D**) dermoscopy after treatment with fractional CO2 laser alone; vellus hair (6.6%), terminal hair (93.4%), and hair diameter diversity (25.4%) per field. Vellus hair; blue arrow, terminal hair; black arrow, single pilosebaceous unit; green arrow, and peripilar sign; yellow circle

**Fig. 2:**
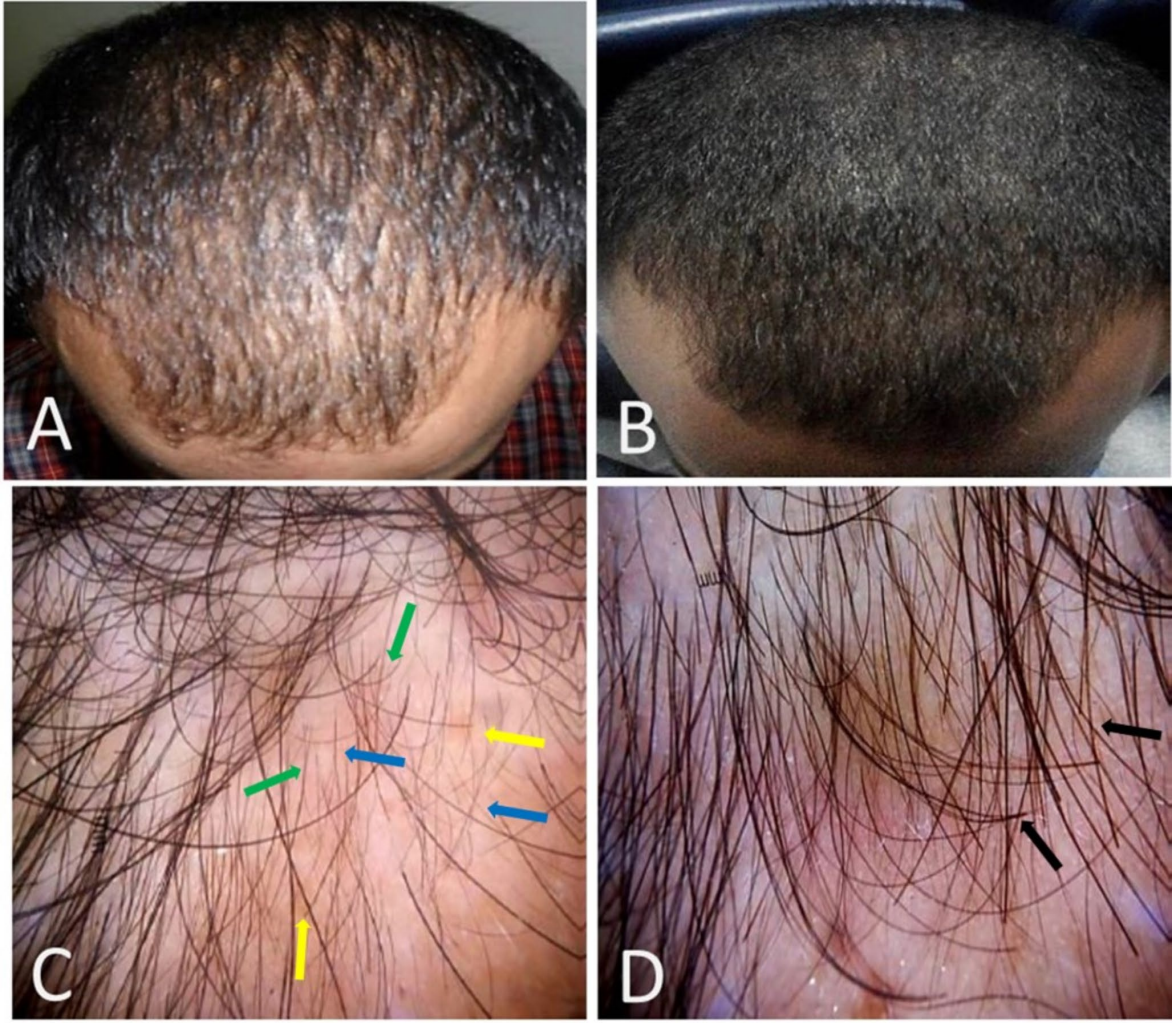
22-year-old male patient, grade I AGA, with a 3-year duration, showing significant improvement (**A**) Clinical photos before treatment (**B**) showing moderate improvement after treatment. (**C**) dermoscopic photos before treatment, counting vellus hair (16.8%), terminal hair (83.2%), and calculating hair diameter diversity (52.1%) per field. (**D**) dermoscopy after treatment with fractional CO2 laser combined with topical dutasteride; vellus hair (13.5%), terminal hair (86.5%), and hair diameter diversity (86.5%) per field. Vellus hair; blue arrow, terminal hair; black arrow, single pilosebaceous unit; green arrow, and yellow dot; yellow arrow

Figures 1 and 2 display clinical and dermoscopic images of patients before treatment and 3 months after the last session.

**Table 4:** Comparison between two groups as regards dermoscopic evaluation after treatment

	Fractional CO2 laser alone					Fractional CO2 laser Plus Topical Dutasteride					*P* value‡
	Mean	SD	Median	Minimum	Maximum	Mean	SD	Median	Minimum	Maximum	
**V after**	24.97	18.55	17.40	6.30	60.00	14.50	6.95	13.80	5.00	29.80	0.161
**T after**	72.15	22.86	82.50	23.10	93.70	85.23	7.13	86.20	70.20	95.00	0.187
**HDD after**	38.81	13.50	35.80	21.40	76.90	31.15	6.71	27.70	22.30	45.30	0.056
**SPSU after**	97.85	2.40	98.40	90.70	100.00	94.33	3.32	96.20	89.50	98.60	< 0.001

Vellus hair (V), terminal hair (T), hair diameter diversity (HDD), single pilosebaceous unit (SPSU)

P-value > 0.05: Non-significant; P-value < 0.05: Significant; P-value < 0.01: Highly significant

‡: Mann-Whitney test

**Fig. 3:**
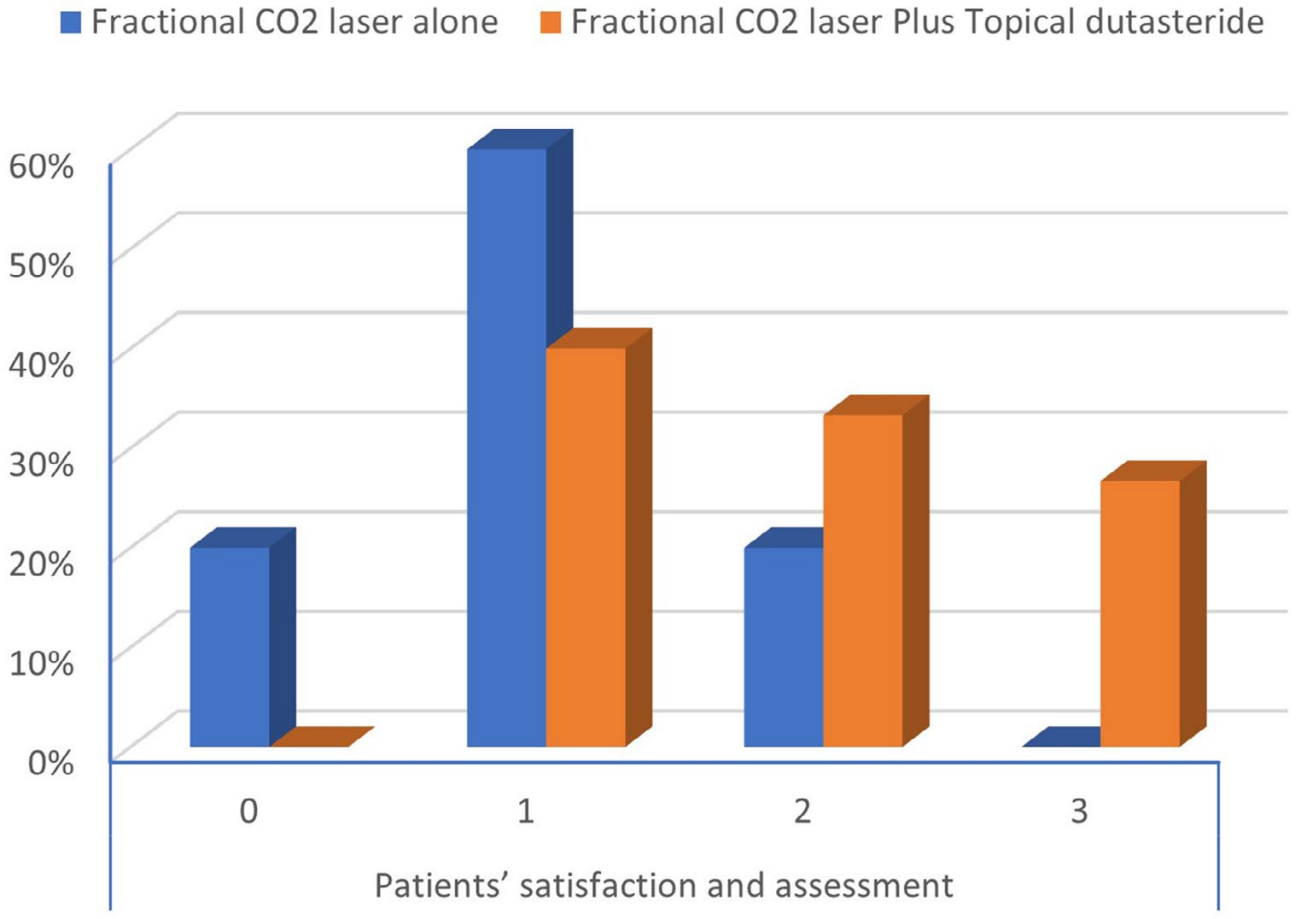
Comparison between two groups as regards patients’ satisfaction

On comparing the degree of improvement between both groups according to the investigator’s global assessment, excellent (+ 3) and good improvement (+ 2) were observed in 6.7% and 40.0% of patients, respectively, in the laser plus dutasteride group, while none of the patients in the laser only group showed excellent or good improvement. Poor improvement (+ 1) and no change (0) were observed in 66.7% and 33.3% of patients, respectively, in the laser-only group compared to 33.3% and 20.0% of patients, in the laser plus dutasteride group. A statistically significant difference was found between both groups regarding the degree of improvement, with a p-value of 0.014. (Table 5). Patients treated with fractional CO2 laser plus dutasteride showed a better degree of satisfaction than patients treated with fractional CO2, with a statistically significant difference between both groups (p-value = 0.040) (Fig. 3).

**Table 5:** Comparison between two groups as regards patient’s degree of improvement

		Fractional CO2 laser alone		Fractional CO2 laser Plus Topical Dutasteride		P value*
		Count	%	Count	%	
**photographic assessment** (investigator’s global assessment)	**0**	5	33.3%	3	20.0%	0.014
	**1**	10	66.7%	5	33.3%	
	**2**	0	0.0%	6	40.0%	
	**3**	0	0.0%	1	6.7%	

P-value > 0.05: Non-significant; P-value < 0.05: Significant; P-value < 0.01: Highly significant

*: Chi-square test

In the group treated with fractional CO2 laser alone, there was no significant relationship between improvement (photographic assessment) and age, duration of AGA, family history, alopecia grading, or dermoscopic findings before treatment.

**Fig. 4:**
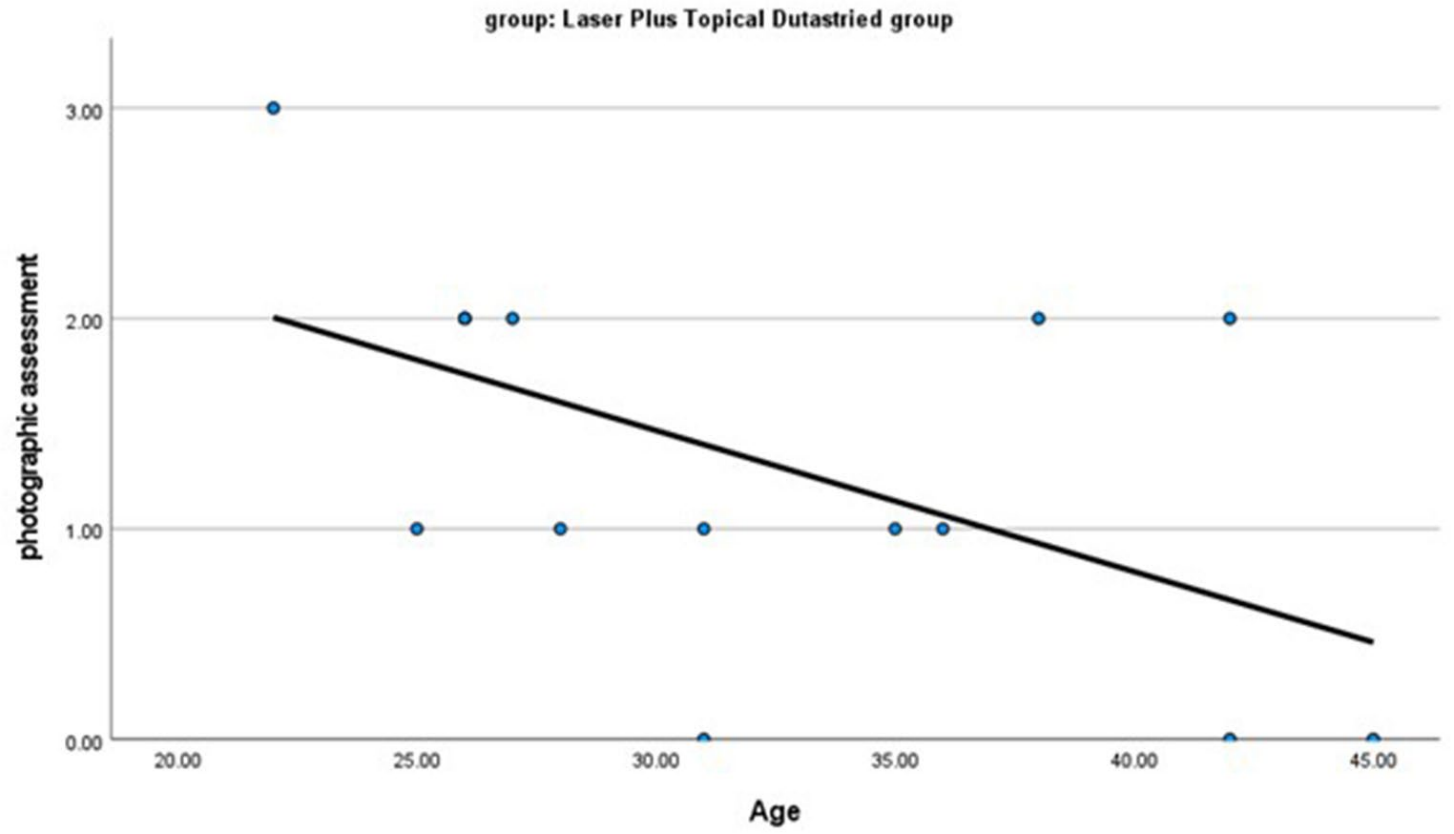
There is a significant negative correlation between photographic assessment and the age of the patient. *r* = -0.531 and *p* = 0.042

**Fig. 5:**
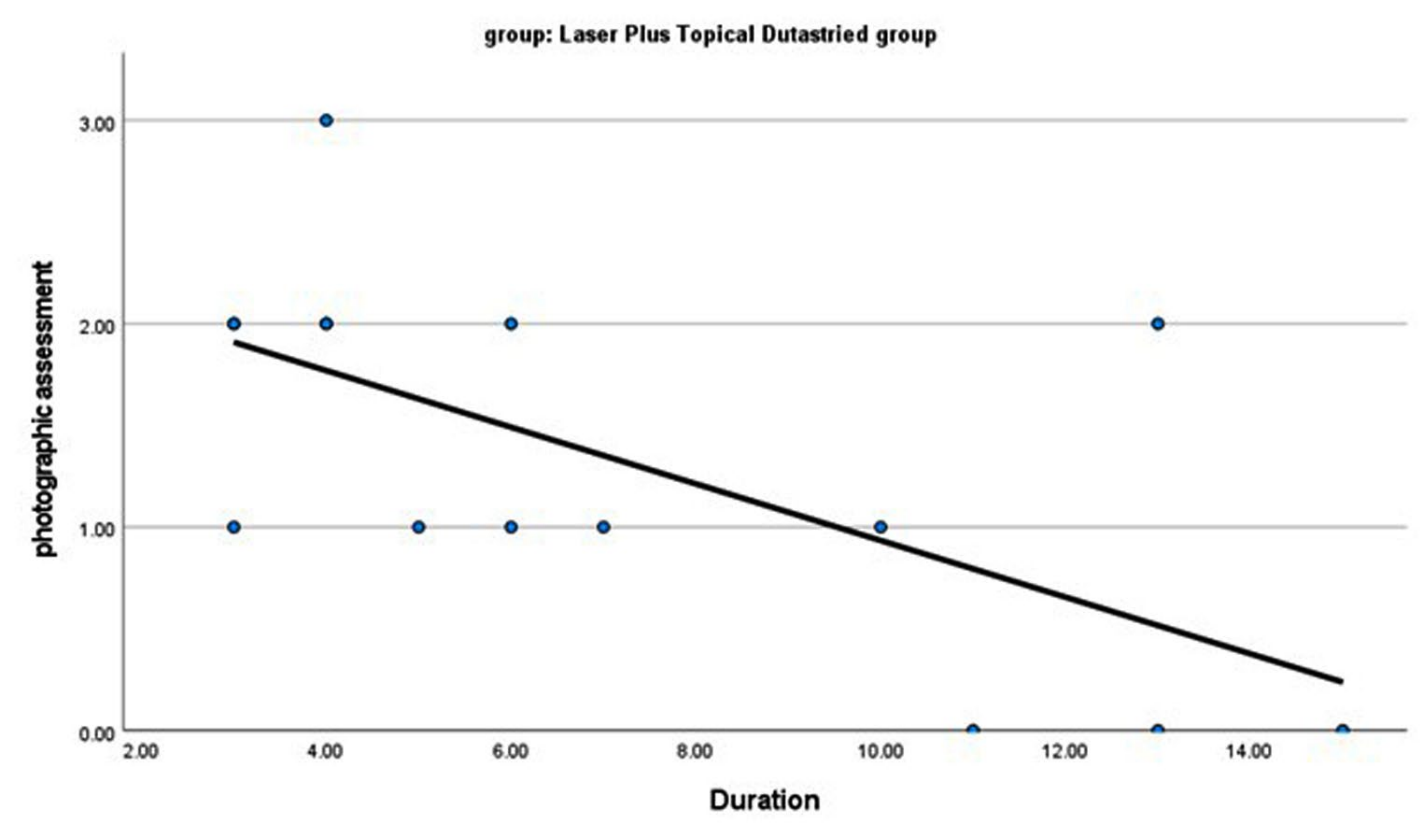
There is a significant negative correlation between photographic assessment and the duration of androgenic alopecia. *r* =-0.606 and *p* = 0.017

In the group treated with fractional CO2 laser combined with topical dutasteride, there was a significant negative correlation between improvement (photographic assessment) and age as well as duration of AGA (*r* = -0.531, *p* = 0.042 and *r* = -0.606, *p* = 0.017 respectively). (Figures 4 and 5), while there were no significant relations between improvement (photographic assessment) and family history, alopecia grading, or dermoscopic findings before treatment.

**Fig. 6:**
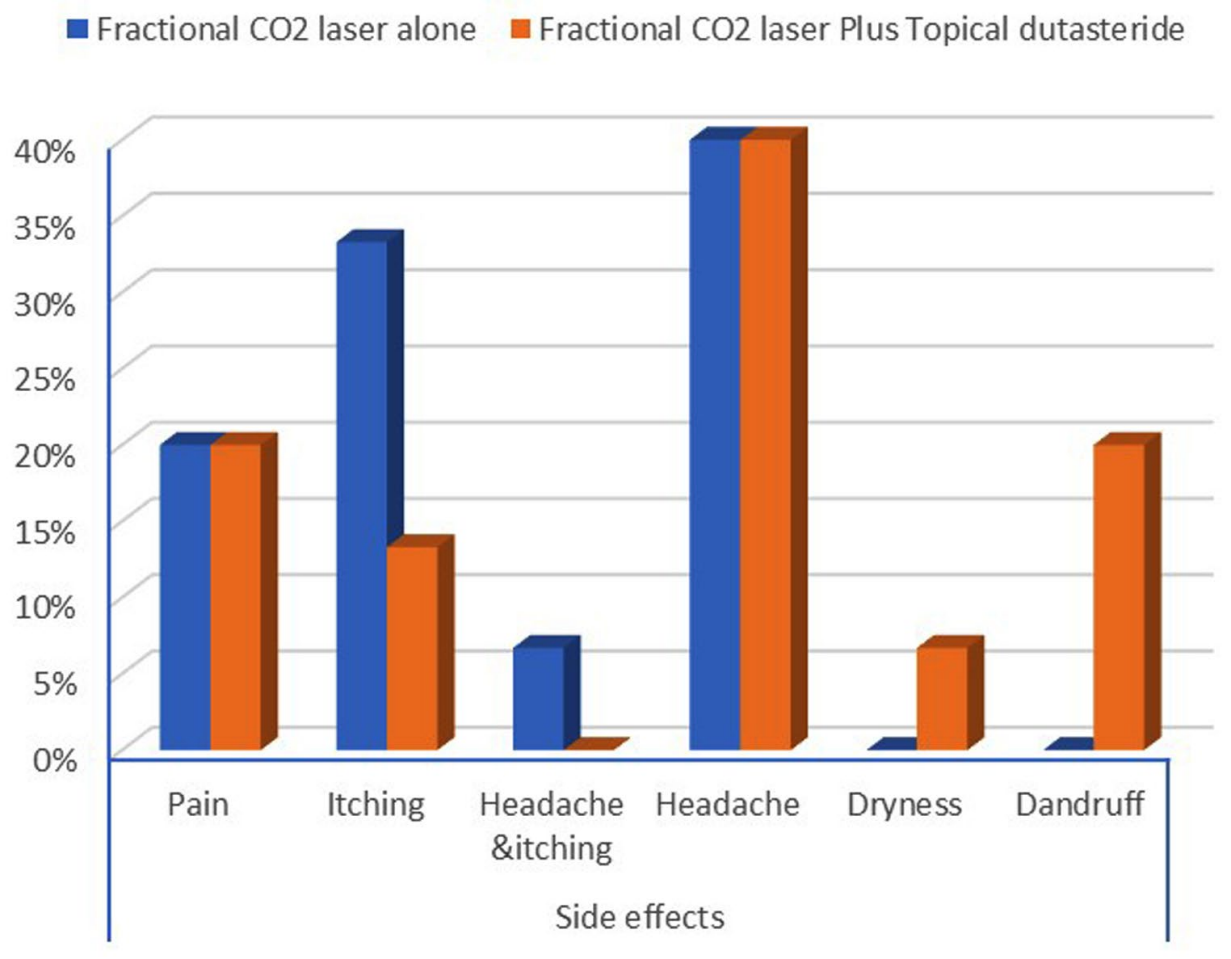
Comparison between two groups as regards patient’s side effects

The reported side effects in both groups were mild and transient in the form of mild pain, headache, dryness, and itching, without a statistically significant difference between the two groups (p-value = 0.305) (Fig. 6).

## Discussion

AGA is the most common cause of male baldness. No medication is effective in eradicating the condition [5].

The paradoxical hair growth that appears as a complication during laser hair removal treatment brought attention to the use of the laser in inducing hair growth. Also, low-level light therapy, including the excimer laser, showed the ability of light to treat alopecia [7].

Fractional lasers have been studied as treatment options for AGA in a few studies. On the other hand, no study has evaluated the efficacy of fractional CO2 laser with topical dutasteride in the treatment of male AGA. Based on these, we conducted this study on 30 male Egyptian patients ranging in age from 22 to 45 years to evaluate the efficacy of fractional CO2 laser at 10,600 nm versus fractional CO2 laser with topical dutasteride in the treatment of male AGA.

Dermoscopic findings after treatment in the laser-only group showed a statistically significant decrease in vellus hair, diameter diversity, peripilar sign, and single pilosebaceous unit compared to findings before treatment. There was a statistically significant increase in terminal hair after treatment compared to before treatment.

This improvement may be related to the fact that fractional lasers produce multiple microthermally damaged areas, which are called microscopic treatment zones (MTZ) with equal depth, diameter, and density. This initiates a rapid healing process [11]. Re-epithelialization appeared in the first 24 h with the migration of keratinocytes from the adjacent normal tissue of the microthermal zones. Dermal collagen remodeling responses persist for at least 3 months after fractional laser treatment [12]. Wound healing enhances growth factor production and has a stimulant impact on dermal papillae and stem cells [13]. There is a relationship between wound healing and hair follicles. Hair follicles promote a normal healing process. On the other hand, hair follicles may arise after wounding. Some studies on rabbits, mice, and even humans showed that hair follicles developed following wounding [12].

Although the mechanisms of hair regrowth induced by fractional lasers of different types are not fully known, it has been revealed that fractional lasers induce Wnt and β-catenin expression, pathways that are involved in hair growth. The microenvironment of healing wounds and the increase in vascularity may play a role in stimulating the stem cells of the hair follicles, causing anagen induction [14].

Our results agreed with Salah et al.‘s [2] study, which reported a significant increase in the number of thick hairs after treatment of male AGA with ablative fractional CO2 alone and combined with minoxidil. Unfortunately, there were few studies about fractional CO2 lasers in AGA treatment. Kim et al. [15] conducted trials with a 1,550-nm fractional erbium-glass laser on males with AGA, and an increase in hair density was observed in the majority of the participants. These results were in agreement with our findings.

Regarding the laser plus topical dutasteride group in our study, we have detected a statistically significant decrease in vellus hair, diameter diversity, peripilar sign, and single pilosebaceous unit after treatment compared to before treatment. There was a statistically significant increase in terminal hair after treatment compared to before treatment.

Ablative laser therapy could help with the effective delivery and uniform distribution of topical agents to the dermis, the site of most of the hair [8]. It generates vertical channels in the epidermis and dermis while saving the surrounding tissue, allowing for easy access for drugs to the dermis [12].

In agreement with our results, Bertin et al. [13], who used a non-ablative 1550 nm fractional erbium glass laser (8 sessions at 7 mJ, density 120 MZT/cm2) as assisted drug delivery of topical finasteride and topical growth factors, showed improvement in hair density and hair regrowth.

There was no statistically significant difference between both groups regarding dermoscopic signs before and after treatment except for the single pilosebaceous unit, which showed a statistically significant reduction in the laser plus topical dutasteride group compared to the laser-only group.

These results agreed with Salah et al.‘s [2] study, in which the hair thickness (thin and thick) significantly increased after the treatment with ablative fractional CO2 laser alone or in combination with minoxidil without a statistically significant difference between both groups. Only the number of single pilosebaceous units significantly changed after the treatment in the combined group compared to the laser-only group.

According to the global photo assessment in our study, a statistically significant improvement in hair growth was found in the fractional CO2 laser plus topical dutasteride group compared to the laser-only group. This could be attributed to the synergistic effects of combined dutasteride with fractional CO2 lasers in promoting hair growth [14], in addition to the role of fractional CO2 lasers in enhancing the homogenous delivery of dutasteride deep into the dermal layer.

Several studies with different treatment protocols and other combinations suggested that laser-assisted drug delivery (ablative laser fractional photothermolysis) in AGA treatment enhances cutaneous topical delivery of drugs and promotes hair growth [16–18]. Huang et al. [16] used an ablative fractional CO2 laser with hair growth factors; Cho et al. [17] used a fractionated thulium laser with growth factor solutions that gave a more promising effect than the laser alone; and Cohen [18] studied the effect of platelet-rich plasma with ablative laser fractional photothermolysis.

In the group treated with fractional CO2 laser alone, there was no significant correlation between clinical improvement and age, duration of AGA, or grade of AGA. Similar to our finding, Esmat et al. [19] also noticed that there was no significant correlation between the improvement and age or duration of AGA with low-level light therapy.

In the group treated with a fractional CO2 laser and topical dutasteride, there was a significant negative correlation between clinical improvement, age, and duration of AGA. Patients with younger ages and shorter durations of AGA showed better hair growth. This could be attributed to the impact of aging on the stem cells of hair follicles [20]. Moreover, perifollicular fibrosis is increased with a longer duration of AGA [21], which may affect the response to treatment. There was no significant relationship between clinical improvement and the grade of AGA or dermoscopic findings.

Transient hair shedding during the session was observed as a side effect in all of our patients. Slight pain, headache, itching, and erythema appear at different degrees in our patients, with no statistically significant difference in either group. headache and itching were reported in some patients, which were tolerated. This agreed with Cho et al.‘s [17] trial on patients with various types of hair disorders, including scarring alopecia, treated with ablative fractional laser therapies, which showed that lasers enhance hair regrowth with nearly no side effects.

Also, Kim et al. [15] reported that some of the participants complained of mild post-treatment erythema, pruritus, dryness, dandruff, and transient hair shedding in patients treated with a 1,550-nm fractional erbium-glass laser.

The present study’s limitation was the exclusion of female patients with AGA. Although this study focused only on male androgenic alopecia, fractional CO2 laser therapy, both alone and in combination with topical dutasteride, could be beneficial for female patients with androgenic alopecia. However, females were excluded, as the potential for hair cutting during laser sessions makes it challenging to use on females. Additionally, there is also increased epithelial thermal injury and downtime.

In conclusion, this study suggests that fractional laser therapies have a positive effect on hair regrowth, either alone or as laser-assisted drug delivery. Ablative fractional CO2 laser combined with other topical agents, such as dutasteride, may provide a safe alternative treatment for AGA.

Based on our findings, we recommend combination therapy specifically for patients with moderate-to-advanced AGA (Norwood-Hamilton scale grades III-V), as these patients showed greater improvements in hair density and scalp coverage when treated with both modalities compared to CO₂ laser alone. Additionally, there is a need to extend the follow-up period for the possibility of recurrence.
